# Arbuscular mycorrhizal fungi enhanced rice proline metabolism under low temperature with nitric oxide involvement

**DOI:** 10.3389/fpls.2022.962460

**Published:** 2022-09-28

**Authors:** Zhilei Liu, Shiting Bi, Jingrou Meng, Tingting Liu, Pengfei Li, Cailian Yu, Xianlong Peng

**Affiliations:** ^1^ College of Resources and Environment, Northeast Agricultural University, Harbin, China; ^2^ Key Laboratory of Germplasm Innovation, Physiology and Ecology of Grain Crop in Cold Region (Northeast Agricultural University), Ministry of Education, Harbin, China; ^3^ The School of Material Science and Chemical Engineering, Harbin University of Science and Technology, Harbin, China

**Keywords:** arbuscular mycorrhizal fungi (AMF), low temperature, nitric oxide (NO), proline, nitrogen

## Abstract

Arbuscular mycorrhizal fungi (AMF) are known to improve plant stress tolerance by regulating proline accumulation, and nitric oxide (NO) plays an important signaling role in proline metabolism. Environmental nitrogen (N) affects AMF colonization and its contribution to host plants resistance to stress conditions. However, the relationship between proline metabolism and NO in mycorrhizal rice and the effect of N application on symbiont proline metabolism under low temperature have not been established. Pot culture experiments with different temperature, N and exogenous NO donor treatments were conducted with non-mycorrhizal and mycorrhizal rice. The results showed that AMF enhanced rice proline accumulation under low-temperature stress and decreased glutamate (Glu) and ornithine (Orn) concentrations significantly. In comparison with non-mycorrhizal rice, AMF colonization significantly decreased the Glu concentration, but had little effect on the Orn concentration under low-temperature stress, accompanied by increasing expression of *OsP5CS2*, *OsOAT* and *OsProDH1*. Exogenous application of NO increased proline concentration both under normal and low temperature, which exhibited a higher increase in mycorrhizal rice. NO also triggered the expression of key genes in the Glu and Orn pathways of proline synthesis as well as proline degradation. Higher N application decreased the AMF colonization, and AMF showed greater promotion of proline metabolism at low N levels under low temperature stress by regulating the Glu synthetic pathway. Meanwhile, AMF increased rice nitrate reductase (NR) and nitric oxide synthase (NOS) activities and then enhanced NO accumulation under low N levels. Consequently, it could be hypothesized that one of the mechanisms by which AMF improves plant resistance to low-temperature stress is the accumulation of proline *via* enhancement of the Glu and Orn synthetic pathways, with the involvement of the signaling molecule NO. However, the contribution of AMF to rice proline accumulation under low-temperature stress was attenuated by high N application.

## Introduction

Low-temperature stress is an important abiotic factor that adversely affects the growth and productivity of plants globally (Sanghera et al., 2011; Ermilova, 2020). By affecting cellular macromolecules, such as DNA, RNA and proteins, and membranes, low-temperature stress could lead to slower metabolism, cell membrane rigidification, the loss of membrane function, and ultimately reduced crop productivity (Apel and Hirt, 2004; Obata and Fernie, 2012). Arbuscular mycorrhiza (AM), ubiquitous symbiotic associations established between arbuscular mycorrhizal fungi (AMF) and higher plant roots, can be found in 70–90% of terrestrial plant species (Parniske, 2008). Fungal symbionts obtain energy from organic compounds synthesized by plants, and the plants benefit from enhanced nutrient and water uptake, increasing metabolism and aboveground productivity (Bago et al., 2000; Smith and Smith, 2011). In addition, AMF can alleviate the damage of abiotic stresses on plants, such as cold, drought, salinity, heavy metal stress, and biotic stresses (Marulanda et al., 2003; Liu et al., 2015; Begum et al., 2019; Feng et al., 2020; Hao et al., 2022).

The synthesis of osmoprotectants, including soluble sugars, proline, polyamines, betaines, and acylated sterols, can improve plant resistance to low-temperature stress (Foyer et al., 2002; Theocharis et al., 2012). Proline, an organic osmoregulatory substance in plant cells, plays important roles in maintaining the osmotic balance between protoplasm and the environment, preventing water loss, enhancing protein hydration, maintaining membrane structural integrity, and mitigating the damage from low-temperature stress to cells (Macarthur and Thornton, 1991; Kavi Kishor et al., 2015). In general, there are two pathways for the synthesis of plant proline: the glutamate (Glu) pathway and the ornithine (Orn) pathway, with Glu and Orn as the initial substrates. Δ1-Pyrroline-5-carboxylate synthetase (P5CS) and glutamate dehydrogenase (GDH) are the core enzymes of the Glu pathway, and ornithine transaminase (OAT) and arginase are the key enzymes of the Orn pathway. Meanwhile, proline dehydrogenase (ProDH) is the major rate-limiting enzyme for proline degradation (Kishor et al., 2005; Qamar et al., 2015). There is great accumulation of proline under the circumstance of low-temperature stress and widely reports for the contribution of proline in alleviating cold injury has been studied in rice, soybean, maize, etc. (Verbruggen and Hermans, 2008). The P5CS and GDH are two key enzymes in proline synthesis under adverse stress, with P5CS being the rate-limiting enzyme (Liang et al., 2013; Kishor and Sreenivasulu, 2014). Under low-temperature stress, plants may relieve the feedback inhibition of OAT by proline and enhance proline accumulation, thereby increasing plant resistance to stress as well (Ruiz et al., 2002). It has also been suggested that drought and low temperature activate both pathways of proline synthesis (Yang et al., 2015).

There’s a fair amount of research that suggests that AMF increased host plant stress resistance through increasing proline accumulation, improving osmotic adjustment in coping with low temperature, drought, flooding, and salinity etc. (Zhu et al., 2010; Chen et al., 2014a; Chun et al., 2018; Al-Arjani et al., 2020). Under salt stress conditions, mycorrhizal plants increased synthesis of proline based in a diminishing Na^+^ compared to non-mycorrhizal plants (Santander et al., 2019). The proline content of perennial ryegrass treated with AMF was about three times higher under low temperature stress (Yan et al., 2021). Otherwise, the lower level of proline, P5CS and OAT activity was observed in mycorrhizal plants than in non-mycorrhizal counterparts (Asrar et al., 2012). Similar findings showed that the proline metabolism pathway in mycorrhizal symbionts under drought stress in certain plants displayed different results in which proline accumulation in mycorrhizal plants occurred mainly through the regulation of the Glu pathway rather than the Orn pathway (Zou et al., 2013). However, the synthesis and degradation pathway of host plants affected by AMF colonization under low temperature was still little known, which need to further study.

Nitric oxide (NO) is considered to be an important signaling molecule involved in various plant responses to biotic and abiotic stresses (Arasimowicz and Floryszak-Wieczorek, 2007). Previous studies have suggested that NO can regulate the proline biosynthetic pathway in plants *via* enzymatic and transcriptional modulation (Filippou et al., 2013). Under low-temperature stress, NO has been found to affect plant proline accumulation by increasing P5CS activity and decreasing ProDH activity (Wang et al., 2013; Koenigshofer and Loeppert, 2019). However, these results also indicated that NO could promote proline accumulation by upregulating the Orn pathway (Wang et al., 2016b). Interestingly, mycorrhizal plants under low-temperature stress have been found to exhibit greater concentrations of NO in rice roots (Liu et al., 2013). In mycorrhizal symbiosis, NO has been proposed to be involved in the activation of the nodule formation and symbiotic nutrient communication (Calcagno et al., 2012; Wang et al., 2013; Hu et al., 2021). However, the role of NO and the mechanism underlying the promotion of plant proline metabolism by AMF under low-temperature stress remain puzzling.

The promotion of host plant nutrient uptake by AMF is critically determined by nutrient, such as phosphorus (P) and nitrogen (N) levels. The negative effect of P on AM hyphal growth and AMF colonization has been frequently reported (Schroeder and Janos, 2005; Verbruggen et al., 2013), as well as low N levels enhanced AMF colonization (Schmidt et al., 2011; Wang et al., 2013). Only older leaves retained mycorrhizal enhancement whereas for upper or middle maize leaves the difference between mycorrhizal and non-mycorrhizal plants was not significant statistically under moderate or high NPK fertilization rates (Polcyn et al., 2019). The contribution of AMF was also affected by N status under low temperature stress. AMF enhance the tolerance of the host plant to low temperature by promoting the N and carbon (C) metabolism on the plant side, while AMF having a greater promotion during this process in addition to improving the growth of the host plant under low N level (Liu et al., 2013). In general, there exists positive correlation between N availability and proline accumulation (Claussen, 2002). Thus, it can be speculated that the regulation of host plant proline metabolism by AMF under low temperature stress could be also related to N levels. However, little research was conducted before on this aspect.

To investigate the abovementioned questions, two experiments were conducted with potted rice (*Oryza sativa L.*) as the host plant and *Rhizophagus irregularis* as the AMF. Different temperature, exogenous NO donor and N treatments were performed. Physiological parameters, N metabolism enzymes and expression of rice proline metabolism genes, as well as concentrations of proline, Glu and Orn, were measured to test the following three hypotheses: (1) AMF regulate host plant proline metabolism *via* either the Glu and Orn pathway or through both pathways under low temperature stress; (2) NO not only acts as a signaling molecule promoting symbiotic mycorrhizal formation but is also involved in the regulation of rice proline metabolism by AMF; (3) environmental N level affects mycorrhizal proline metabolism under low temperature stress by regulating NO synthesis. The results of this study could provide more insight into the mechanism of AMF improving plant resistance to low temperature stress.

## Material and methods

### Soil materials and AMF colonization

Plants were grown in 2-kg growth substrate pots in a mixture of black soil, sand and perlite (1:1:1, v/v/v). The perlite was added to increase the porosity of the mixture. Each pot contained 111 mg kg^-1^, 33.5 mg kg^-1^ and 111 mg kg^-1^ available N, P and K, respectively. The soil was autoclaved for 2 h at 121°C and then placed in pots that had been sterilized with 75% alcohol.

Half of the plants received 25 g inoculum in the form of dry soil and *Medicago truncatula* roots colonized by *Rhizophagus irregularis* (BGC BJ09) provided by the Institute of Plant Nutrition and Resources, Beijing Academy of Agriculture and Forestry in China. The corresponding non-mycorrhizal plants received 10-mL washings from the inoculum filtered through Whatman 3M paper filters to obtain a similar composition of microorganisms except for the mycorrhizal fungus.

### Plant growth conditions

Uniform, full rice seeds were submerged in 0.5% NaClO and 75% alcohol for 5 min and 30 min, respectively, for sterilization. The seeds were rinsed three times with double-distilled water and then germinated on wet filter paper Petri dishes in the dark at 30°C ± 2.0°C. Next, the 2-day-old seedlings were transferred to a hydroponic culture system containing 0.5× concentration International Rice Research Institute (IRRI) nutrient solution at pH 5.5 and grown in a growth chamber at 20/28°C ± 2.0°C (night/day) for 7 d before being transplanted into pots. The chamber was set to a 16-h light/8-h dark cycle at 22°C ± 1.0°C with light provided by fluorescent lamps at 20000 lux light intensity. Six uniform rice seedlings were transplanted into separate pots after 7 days of cultivation, and the pots were arranged randomly in a block design outside. Approximately 100 mL IRRI nutrient solution, which was modified to contain 75% of the normal concentration of P to create P conditions for mycorrhizal fungal colonization, was provided every other day for each pot.

### Experiment 1

To identify the effect of AMF on rice proline metabolism under low temperature stress and the role of NO on mycorrhizal rice proline metabolism, different temperature and exogenous NO application treatments were established. At the panicle initiation stage (corresponding code BBCH 30) (Meier, 2001), which is the stage in which rice are most sensitive to low temperature, non-mycorrhizal and mycorrhizal rice were subjected to low-temperature stress combined with exogenous NO treatment. Two temperature treatments, 15°C ± 1.0°C and 22°C ± 1.0°C, were applied to non-mycorrhizal and mycorrhizal rice, and the NO donor sodium nitroprusside (SNP) was applied at two concentrations (0 and 100 μM) to the non-mycorrhizal and mycorrhizal rice grown at the two temperatures. Samples were treated with 20 mL SNP per pot every day. Meanwhile, the pots were watered every other day with IRRI nutrient solution and maintained for 7 d before harvest. The chamber parameters were set identically to those described above. Five biological replicates were performed for each treatment using a completely randomized design.

### Experiment 2

To characterize differences in mycorrhizal rice proline metabolism under different N concentrations at low temperature, a pot culture experiment was conducted with two N levels and two temperatures. Six uniform 7-day-old seedlings were transplanted into separate pots; half of the mycorrhizal and non-mycorrhizal pots were supplied with approximately 100 mL IRRI nutrient solution at a 30 mg N L^-1^ (N1) concentration, and the other pots were supplied at 80 mg N L^-1^ (N2). The nutrient solution was provided every other day for each pot. At the panicle initiation stage, two temperatures, 15°C ± 1.0°C and 22°C ± 1.0°C, were applied to the non-mycorrhizal and mycorrhizal rice growing under the two N conditions. The chamber parameters were set the same as those described above, and rice seedlings were harvested after 7 days of treatment. Five biological replicates were performed for each treatment using a completely randomized design.

### Mycorrhizal colonization rate and plant biomass measurement

Arbuscular mycorrhizal colonization rate was calculated according to Phillips and Hayman (1970). Rice root segments were cut into 1.0-cm pieces and stained with trypan blue. Mycorrhizal colonization rate was then determined according to (Trouvelot et al., 1986). Colonization was expressed as the colonization frequency (F %) in the root system.

After harvesting, the samples were rinsed with distilled water and dried with filter paper. The plant height, root lengths of the rice plants were measured, and the samples were kept in an oven at 105°C for 30 min and 85°C to obtain the dry weight. The measure of the mycorrhizal responsiveness (MR) included the mycorrhiza-induced increases in shoot growth (MSR) and root growth (MRR) and the mycorrhiza-induced increases in the root N content (MNR). MR was calculated according to Chu et al. (2013): MR = M - NM, where M and NM refer to the plant height or root length of mycorrhizal and non-mycorrhizal plants, respectively.

### NO detection

NO content was measured using the method of Hu et al. (2003). A 0.6-g sample of fresh tissue from each sample was added to 3 mL of precooled 50 mM acetate buffer (pH 3.6, containing 4% zinc diacetate) and ground into a homogenate at 4°C, followed by centrifugation at 9500 × g for 15 min. The supernatant was collected, and 1 mL of the pellet was washed with acetate buffer and centrifuged twice as above. The supernatants were combined, and 0.1 g of activated charcoal was added, followed by vortexing and filtration. A 1-mL aliquot of the filtrate was added to 1 mL of Griess reagent (1% sulfanilamide/0.1% N-(1-naphthyl)-ethylenediamine dihydrochloride in 5% phosphoric acid). The mixture was incubated at room temperature for 30 min, and the absorbance was measured at 540 nm.

### Enzymatic assays

The nitrate reductase (NR) activity was measured according to Yu and Zhang (2012), with slight modifications. A 0.1-g sample of fresh tissue was homogenized on ice in 2.0 mL extraction solution containing phosphate buffer (25 mM, pH 7.5, a mixture of K_2_HPO_4_ and KH_2_PO_4_), cysteine (5 mM), and EDTA-Na_2_ (5 mM). The homogenate was centrifuged at 4000 rpm at 4°C for 10 min, and the supernatant was collected. A 0.4-mL aliquot of the enzyme extract was added to 1.6 mL of reaction reagent (1.2 mL of 0.1 M KNO_3_-phosphate buffer and 0.4 mL 2.0 mg mL^-1^ NADH), and the mixture was incubated at 30°C for 30 min. For the control, 0.4 mL phosphate buffer was used instead of 0.4 mL NADH. The reaction was stopped by adding 1.0 mL of 1% 4-aminobenzene sulfonic acid and 1.0 mL of 0.2% 1-naphthylamine and incubated at 30°C for 30 min; the samples were centrifuged at 4000 × g for 10 min, and the absorbance of the supernatant was measured at 540 nm.

The glutamine synthetase (GS) activity was detected according to Yu and Zhang (2012), with slight modifications. A 0.2-g sample of fresh tissue was homogenized in 3.0 mL of Tris-HCl (0.05 M, pH 8.0) in a pre-cooled mortar in an ice bath, followed by extraction with MgSO_4_ (2 mM), DTT (2 mM), and sucrose (0.4 M). The homogenate was centrifuged at 15,000 × g at 4°C for 20 min. A 1.0-mL aliquot of the crude enzyme solution was added to 1.6 mL of reaction mixture containing 0.6 mL of imidazole-HCl buffer (0.25 M), 0.4 mL of sodium hydrogen Glu (0.30 M), and 0.2 mL of MgSO_4_ (0.5 M). The mixture was incubated at 25°C for 5 min; 0.2 mL of hydroxylamine hydrochloride (a mixture of 1 M hydroxylamine hydrochloride and 1 M HCl, 1:1) was added and incubated for 15 min. A 0.8-mL aliquot of FeCl_3_ solution (10% FeCl_3_, 24% trichloroacetic acid and 50% HCl (1:1:1)) was added to terminate the reaction; 1.0 mL of Tris-HCl (0.05 M) instead was used as a control. After centrifugation at 4000 × g for 10 min, the absorbance of the supernatant was measured at 540 nm. The protein content was measured following the method of Bradford (1976), with bovine serum albumin (BSA) as a standard.

The Nitric oxide synthase (NOS) activity was determined following the previous method Shi et al. (2012). About 1 g of samples were ground with liquid N and then resuspended in extraction buffer (50 mM Tris-HCl, pH 7.4, 1 mM EDTA, 1 mM dithiothreitol, 1 mM leupeptin, 1 mM pepstatin, and 1 mM phenylmethylsulfonyl fluoride). After centrifuging at 10,000 × g for 30 min at 4°C, the supernatant was used for NOS activity determination. NOS activity was assayed using t NOS assay kit based on DAF-FM DA (Xiong et al., 2009).

### Analysis of proline, glutamate and ornithine concentrations

Proline concentration in rice was measured according to a method previously described by Shi et al. (2012), with some modifications. Approximately 0.1 g of each rice sample was ground into powder with liquid N and extracted in 3% sulfosalicylic acid. After centrifugation at 12,000 × g for 10 min, the supernatant (2 mL) was mixed with 2 mL of ninhydrin reagent (2.5% (w/v) ninhydrin, 60% (v/v) glacial acetic acid and 40% 6 M phosphoric acid) and 2 mL of glacial acetic acid and incubated at 100°C for 40 min. The reaction was then terminated in an ice bath. The reaction mixture was extracted with 4 mL of toluene, and the absorbance was measured at 520 nm.

Glutamate and ornithine were extracted as described by Salazar et al. (2012) with some modifications. Fresh tissue (0.2 g) from each rice plant was added to separate 125-μL 50% (*v*/*v*) methanol: H_2_O solutions containing isotopically labeled internal standards at 4 μg mL^-1^. Samples were ground in a mixer mill for 60 sec, incubated on dry ice for 5 min, and sonicated in a water bath for 1 min. Two cycles of buffer extraction, grinding, dry ice incubation, and sonication were completed. At the end of each cycle, debris was removed by centrifugation at 10,000 × g and 4°C for 8 min. Extract was transferred each time to a limited-volume vial. Free amino acids were derivatized with the AccQ·Tag Ultra derivatization kit (Waters, Milford, MA, USA). Liquid chromatography of the derivatized amino acids was performed with a Waters AccQ·Tag Ultra column (2.1 × 10 mm, 1.7 μm particles) on a Waters Acquity UPLC system. Eluent A was 10 % AccQ·Tag Ultra concentrate solvent A, and eluent B was 100% acetonitrile. The mobile phase flow rate was 0.7 mL min^-1^, and the sample injection volume was 1 μL.

### Determination of gene expression using quantitative real-time PCR

Total RNA was extracted from frozen rice plants (approximately 100 mg) using Trizol reagent and an RNA Purification Kit (Invitrogen, Carlsbad, CA, USA), including DNase treatment, according to the manufacturer’s protocol. Total RNA was quantified using a spectrophotometer following electrophoresis on a 0.8% (w/v) agarose gel to assess the concentration and integrity of each sample. Approximately 1 µg of total RNA was transcribed into cDNA using Superscript III Reverse Transcriptase (Invitrogen, Karlsruhe, Germany). The quality of the cDNA was assessed by qRT-PCR using primers for the 18S rRNA genes.

qRT-PCR was performed using an Agilent Mx3000P Analyzer (Agilent Technologies Ltd., Santa Clara, CA, USA) in a 15-µL reaction volume containing 1 µL cDNA, 2 µL primer mix, and 7.5 µL SYBR Green Master Mix (Agilent Technologies Ltd., Santa Clara, CA, USA) and 35 cycles were performed. qRT-PCR was performed on three independent biological samples and three technical replicates. Primers for qRT-PCR were designed using Primer Premier software (Version 5.0, PREMIER Biosoft), and all primer sequences are shown in **Table 1**. The comparative ΔΔCT method was used to measure changes in the expression of selected genes relative to untreated controls (Winer et al., 1999).

**Table 1:** List of primers used for real-time PCR.

Gene		Primer sequence (5’-3’)
*OsP5CS1*	Forward	TGTGTACCAACGCGCTATGT
	Reverse	TGTGTACCAACGCGCTATGT
*OsP5CS2*	Forward	GTGGCTTGTGAAGGAGCTGT
	Reverse	TTTGACATGCTTTCGTGCTC
*OsProDH1*	Forward	GCTACTGGGACTTGGGAGTG
	Reverse	GCTACTGGGACTTGGGAGTG
*OsOAT*	Forward	GATTGGGAAAACATACGACCTGAT
	Reverse	AACCGCACTGACAGGAACTACTC
*Os18SrRNA*	Forward	TGTTAATAAAAATCGGTGCGTTGC
	Reverse	AAAACGCAAATGATCAACCGGAC

### Statistical analysis

Statistical analyses were performed using SPSS version 15.0 software. Percentage of root colonization was subjected to one-way ANOVA, other parameters were analyzed by multiway ANOVA, and significant differences between individual means were determined using Duncan’s test at the 5% confidence level based on three biological replicates for each treatment.

## Results

### NO enhanced proline concentrations in non-mycorrhizal and mycorrhizal rice under low temperature

**Figure 1:**
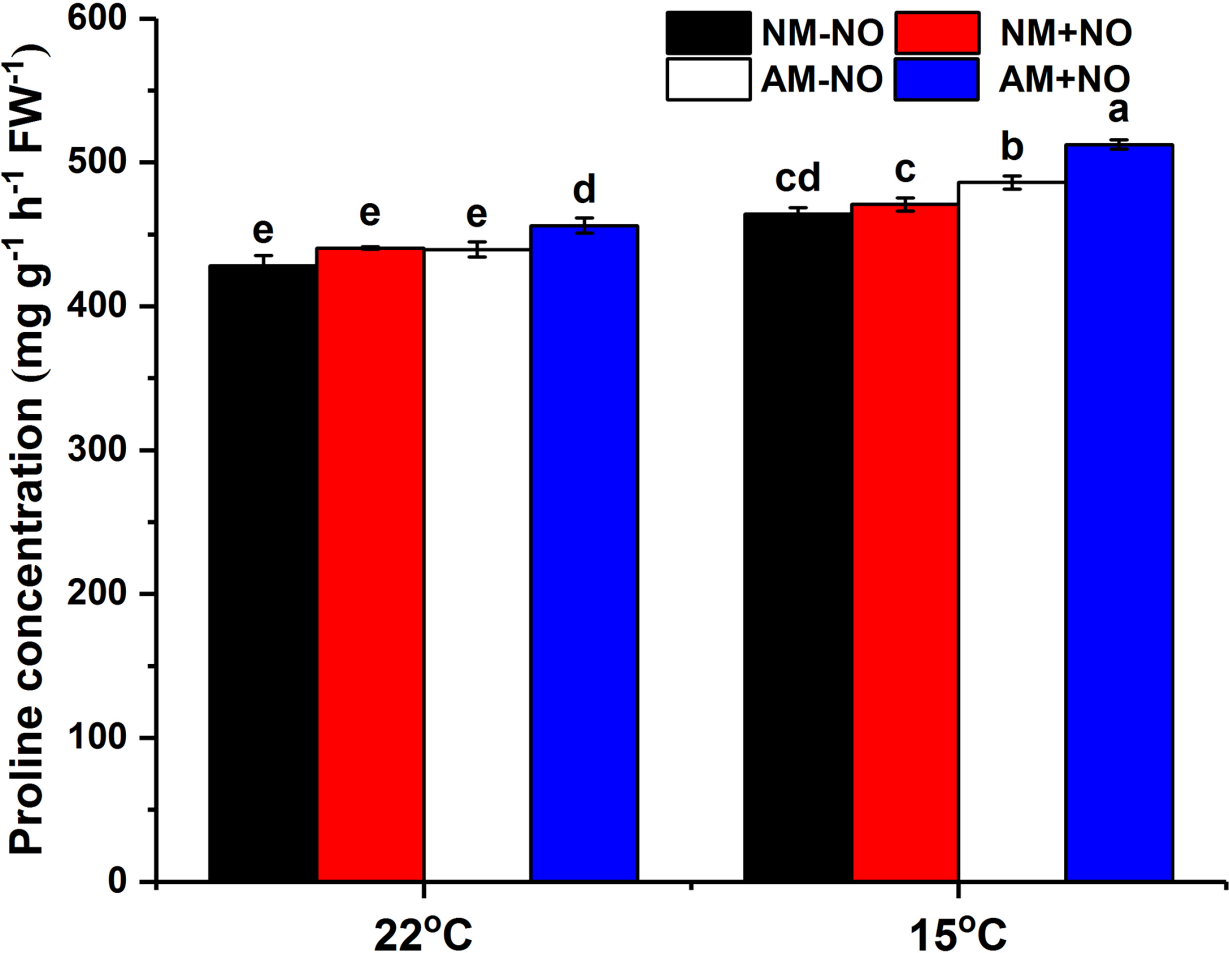
Proline concentration of non-mycorrhizal and mycorrhizal rice treated with different temperatures (25°C and 15°C) and exogenous NO. NM-NO: non-mycorrhizal rice without NO donor, SNP, treatment (*black bars*); NM+NO: NO donor treatment for non-mycorrhizal rice (*red bars*); AM-NO: mycorrhizal rice without NO donor treatment (*white bars*); AM+NO: NO donor treatment for mycorrhizal rice (*blue bars*), where the arbuscular mycorrhizal fungus was *Rhizophagus irregularis.* Values are the means ± standard errors of three biological replicates. Different letters in the column represent significant differences (P<0.05) based on Duncan’s test.

There was no interaction between NO treatment, temperature and arbuscular mycorrhizal fungi, but the main effect was significant. Low temperature led to a rapid accumulation of proline in rice, and AMF enhanced host plant proline accumulation by 4.71% (*P*<0.05) compared to non-mycorrhizal rice under low temperature (**Figure 1**). Exogenous application of NO was able to increase proline concentration not only under normal temperature but also under low temperature conditions, with mycorrhizal rice showing greater increase than non-mycorrhizal rice. Exogenous application of NO elevated the mycorrhizal rice proline concentrations by 3.77% and 5.40% (*P*<0.05) in comparison with pots not treated with NO under normal and low temperature stress, respectively, and increased the AMF colonization rate as well (**Table 2**). Moreover, the NO could enhance plant height and dry weight greater for mycorrhizal symbiont than non-mycorrhizal plant (**Table 2**).

**Table 2:** Effect of NO on rice physiological parameters and arbuscular mycorrhizal colonization rate under different temperature conditions.

Temperature	Treatments	Plant height	Root length	Dry Weight (g·plant^-1^)		Arbuscular mycorrhizal colonization rate (%)
		(cm)	(cm)	Shoots	Roots	
22°C	NM-NO	49.1 ± 0.37^bc^	21.5 ± 0.67^bc^	1.34 ± 0.04^d^	0.61 ± 0.01^ab^	
	AM-NO	50.1 ± 0.73^b^	22.8 ± 0.42^ab^	1.36 ± 0.03^cd^	0.63 ± 0.03^ab^	22.5 ± 1.44^b^
	NM+NO	49.3 ± 0.80^bc^	23.3 ± 0.96^ab^	1.41 ± 0.01^bc^	0.61 ± 0.00^ab^	
	AM+NO	51.9 ± 0.68^a^	24.3 ± 0.59^a^	1.48 ± 0.02^a^	0.66 ± 0.02^a^	28.33 ± 0.83^a^
15°C	NM-NO	46.4 ± 0.47^e^	20.5 ± 0.48^c^	1.27 ± 0.02^e^	0.54 ± 0.01^c^	
	AM-NO	47.7 ± 0.49^cde^	22.3 ± 0.97^abc^	1.34 ± 0.02^d^	0.58 ± 0.03^bc^	20.00 ± 0.72^b^
	NM+NO	47.3 ± 0.14^de^	22.5 ± 0.64^abc^	1.36 ± 0.01^cd^	0.53 ± 0.01^c^	
	AM+NO	48.6 ± 0.08^bc^	23.4 ± 0.28^ab^	1.44 ± 0.01^ab^	0.62 ± 0.01^ab^	25.83 ± 0.42^a^
AMF (A)		**	*	**	**	—
NO		*	**	**	ns	*
Temperature (T)		**	ns	**	**	ns
A × NO		ns	ns	ns	ns	—
A × T		ns	ns	ns	ns	—
NO × T		ns	ns	ns	ns	ns
A × NO × T		ns	ns	ns	ns	—

AMF, arbuscular mycorrhizal fungus. NM-NO: non-mycorrhizal rice without NO donor, SNP, treatment ; NM+NO: NO donor treatment for non-mycorrhizal rice ); AM-NO: mycorrhizal rice without NO donor treatment; AM+NO: NO donor treatment for mycorrhizal rice. Values are means ± standard errors of three biological replicates. Different letters following values within a column represent significant difference (*P* < 0.05) based on Duncan’s test. *P < 0.05, **P < 0.01, ns, not significant.

### Status of proline metabolism in rice receiving exogenous NO application under low-temperature stress

**Figure 2:**
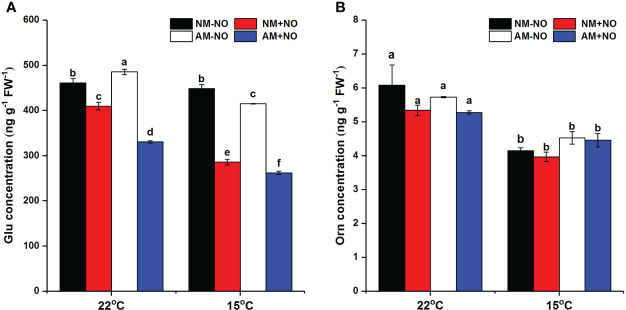
Glutamate (Glu) **(A)** and ornithine (Orn) **(B)** concentrations of non-mycorrhizal and mycorrhizal rice treated with different temperatures (25°C and 15°C) and exogenous NO. Abbreviations are defined in **Figure 1**. Values are the means ± standard errors of three biological replicates. Different letters in the column represent significant differences (P<0.05) based on Duncan’s test.

There was a significant interaction effect of A×N×T on Glu concentration (**Table 3**). To identify the effects of low temperature and NO on proline metabolism in non-mycorrhizal and mycorrhizal rice, the substrate pool for proline synthesis and expression of key proline metabolism genes were analyzed. The results showed that AMF colonization significantly decreased Glu concentrations, but there was little effect on Orn concentrations (**Figure 2**). Low-temperature stress significantly reduced both non-mycorrhizal and mycorrhizal rice Glu and Orn concentrations. Meanwhile, AMF depressed rice Glu concentrations by 16.93% (*P*<0.05) under low-temperature stress, and there was no significant change in non-mycorrhizal rice under low temperature. The exogenous application of NO significantly decreased Glu accumulation in both non-mycorrhizal and mycorrhizal rice, with a greater effect on mycorrhizal rice. However, exogenous application of NO had no effect on Orn concentrations.

**Table 3:** Results of multiway ANOVA for analyses of differences in amino acid concentration and gene expression level in experiment 1.

Factors	Amino acid concentration			Gene expression level			
	Proline	Glu	*Orn*	*OsP5CS1*	*OsP5CS2*	*OsOAT*	*OsProDH1*
AMF (A)	**	**	ns	**	**	**	**
NO	**	**	ns	**	**	**	**
Temperature (T)	**	**	**	*	**	**	*
A × NO	ns	**	ns	ns	**	**	ns
A × T	ns	ns	ns	*	ns	ns	ns
NO × T	ns	**	ns	ns	ns	**	**
A × NO × T	ns	**	ns	ns	ns	**	ns

AMF, arbuscular mycorrhizal fungus. *P < 0.05, **P < 0.01, ns, not significant.

**Figure 3:**
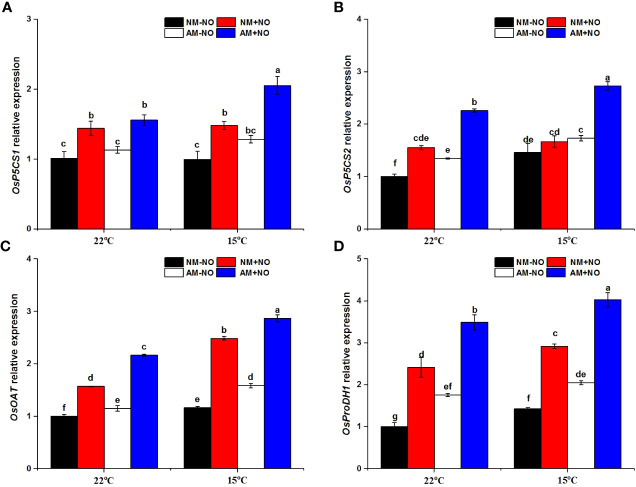
Relative expression levels of *OsP5CS21* **(A)**, *OsP5CS2* **(B)**, *OsOAT* **(C)** and *OsProDH 1* **(D)** in non-mycorrhizal and mycorrhizal rice treated with different temperatures (25°C and 15°C) and exogenous NO. Abbreviations are defined in **Figure 1**. Values are the means ± standard errors of three biological replicates. Different letters in the column represent significant differences (P<0.05) based on Duncan’s test.

There was a significant interaction effect of A×N×T on *OsOAT* gene expression (**Table 3**). Low-temperature stress significantly upregulated the expression of proline synthesis genes (*OsP5CS1*, *OsP5CS2*, *OsOAT*) and degradation genes (*OsProDH1*), and AMF inoculation resulted in higher expression levels than observed in non-mycorrhizal rice under both normal- and low-temperature conditions (**Figure 3**). Exogenous application of NO was able to increase proline metabolism gene expression at normal and low temperatures, and gene expression levels in mycorrhizal rice were again higher than those in non-mycorrhizal rice.

### High N attenuated the promotion of proline metabolism by AMF

**Figure 4:**
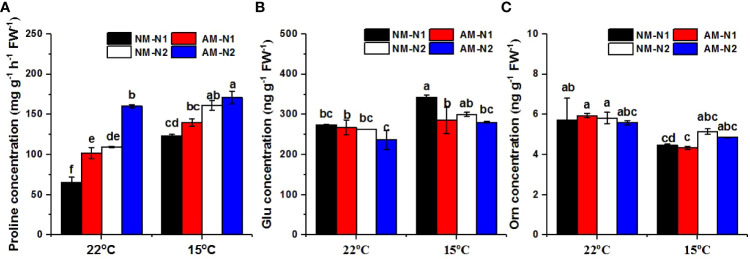
Proline **(A)**, glutamate (Glu) **(B)** and ornithine (Orn) **(C)** concentrations of non-mycorrhizal and mycorrhizal rice treated with different temperatures and N levels. Glu, glutamate; Orn, ornithine; AM and NM represent mycorrhizal and non-mycorrhizal rice, respectively, while the arbuscular mycorrhizal fungus was *Rhizophagus irregularis*. 25°C and 15°C were set. N1: 30 mg N L^-1^, N2: 80 mg N L^-1^. Values are the means ± standard errors of three biological replicates. Different letters in the column represent significant differences (P<0.05) based on Duncan’s test.

N significantly enhanced the accumulation of proline at both normal and low temperatures, and AMF colonization increased proline concentrations as well. However, AMF exhibited greater capability for promotion under low N levels. (**Figure 4A**). Under normal temperature, AMF increased proline concentrations by 55.69% and 46.09% compared to non-mycorrhizal rice under low and high N levels, respectively. Low-temperature stress enhanced the accumulation of proline, and mycorrhizal rice again contained a significantly higher proline concentration under low N level, while there was no significant difference between non-mycorrhizal and mycorrhizal rice under high N level.

There was a significant interaction effect of A×N×T on Glu concentration (**Table 3**). N and AMF had no effect on Glu concentration at normal temperature (**Figure 4B**). However, AMF significantly decreased the Glu concentration at low N level under low-temperature stress; there was no significant difference between mycorrhizal and non-mycorrhizal rice at high N level under low temperature. In addition, N and AMF had little effect on Orn under both normal- and low-temperature conditions (**Figure 4C**).

### NO metabolism under different N and temperature conditions

**Figure 5:**
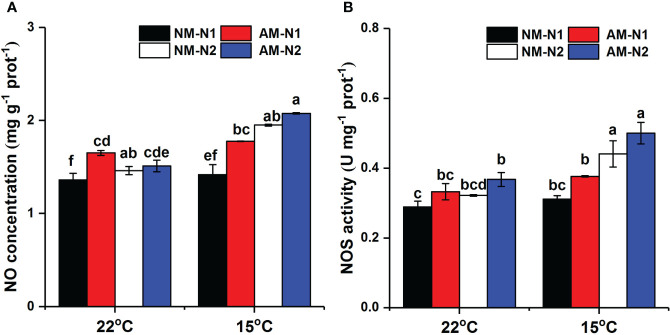
Nitric oxide (NO) **(A)** concentration and nitric oxide synthase (NOS) activity **(B)** of non-mycorrhizal and mycorrhizal rice treated with different temperatures and N levels. Abbreviations are defined in **Figure 4**. Values are the means ± standard errors of three biological replicates. Different letters in the column represent significant differences (P<0.05) based on Duncan’s test.

AMF significantly increased NO accumulation under low N level at normal temperature, and there was no significant difference between mycorrhizal and non-mycorrhizal rice under high N level (**Table 4**). Low temperature significantly increased the NO concentration at the high N level, while the NO concentration was little affected by AMF under high N condition (**Figure 5A**). AMF increased NO concentration by 25.26% (*P*<0.05) at low N level under low-temperature stress. However, AMF had no effect on NOS activity under different N and temperature conditions (**Figure 5B**).

**Table 4:** Results of multiway ANOVA for analyses of differences in amino acid concentration, NO concentration and enzyme activities in experiment 2.

Factors	Amino acid concentration			NOS activity	NO concentration	NR activity	GS activity
	Proline	Glu	Orn				
AMF (A)	**	**	ns	**	**	**	**
Nitrogen (N)	**	**	ns	**	**	**	**
Temperature (T)	**	**	**	**	ns	**	**
A × N	**	**	ns	ns	ns	ns	ns
A × T	ns	ns	ns	ns	ns	ns	ns
N × T	ns	**	ns	**	ns	**	ns
A × N × T	ns	*	ns	ns	ns	ns	ns

AMF, arbuscular mycorrhizal fungus. *P < 0.05, **P < 0.01, ns, not significant.

### Effects of N and low temperature on rice N metabolism enzyme activity

**Figure 6:**
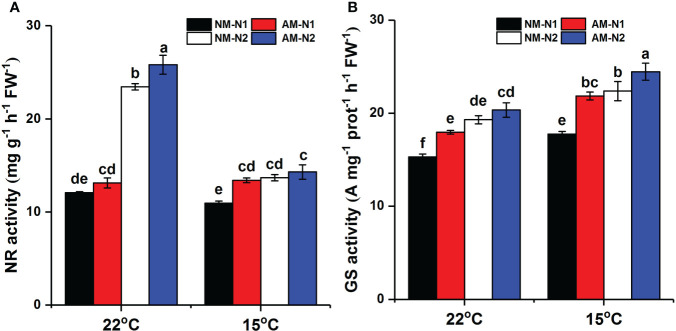
NR **(A)** and GS **(B)** activity of non-mycorrhizal and mycorrhizal rice treated with different temperatures and N levels. Abbreviations are defined in **Figure 4**. Values are the means ± standard errors of three biological replicates. Different letters in the column represent significant differences (P<0.05) based on Duncan’s test.

Increasing N concentration could enhance activity of NR and GS under both normal and low temperatures (**Figure 6**). The promotion of rice NR and GS by AMF was affected by N. At normal temperature, AMF significantly increased NR activity under high N level but showed little effect under low N level. However, the effect of AMF on NR activity exhibited an opposite trend under low-temperature stress. Mycorrhizal rice growing under low N level had 22.42% (*P*<0.05) higher NR activity than did non-mycorrhizal rice, while there was no significant difference between mycorrhizal and non-mycorrhizal rice growing under high N level. In addition, mycorrhizal rice had higher GS activity than did non-mycorrhizal rice at low N level under normal temperature, with a lower effect at high N level. Under low-temperature stress, AMF increased GS activity by 23.01% (*P*<0.05) and 9.27% (*P*<0.05) under low and high N levels, respectively.

### Growth parameters and mycorrhizal colonization rates under different N and low temperature conditions

**Figure 7:**
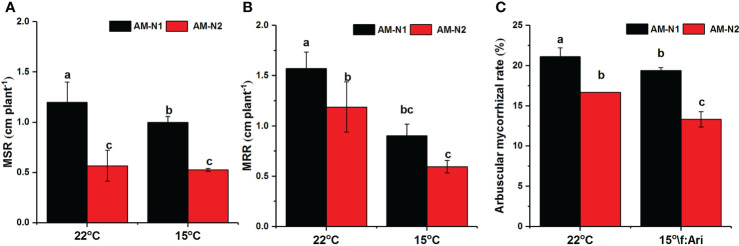
Mycorrhiza-induced increases in shoot growth (MSR) **(A)**, root growth (MRR) **(B)** and mycorrhizal colonization **(C)** for rice under different N and temperature conditions. Abbreviations are defined in **Figure 4**. Values are the means ± standard errors of three biological replicates. Different letters in the column represent significant differences (P<0.05) based on Duncan’s test.

The colonization of AMF increased rice plant height and root length under both normal and low-temperature stress (data not shown). However, AMF had higher MSR and MRR at low N level than at high N level (**Figures 7A, B**). AMF exhibited higher colonization rate under low N condition, and 15°C temperature seemed to have little effect on mycorrhizal colonization rate (**Figure 7C**).

## Discussion

Proline is a well-known osmotolerant and cryoprotectant that has been found to accrue in response to most abiotic stresses, including low temperature (Kishor et al., 2005; Theocharis et al., 2012). A great deal of evidence has shown that mycorrhizal plants accumulate higher root and leaf proline contents than do non-mycorrhizal plants under low-temperature stress (Zhu et al., 2010; Chen et al., 2014a). NO is considered to be an important signaling molecule involved in proline accumulation and mycorrhizal symbiont establishment (Calcagno et al., 2012; Wang et al., 2016a). However, the relevance of deeper mechanisms underlying the action of NO as a signaling molecule in AMF affecting host plant proline metabolism and the effect of N level on mycorrhizal proline metabolism remain to be demonstrated.

Proline accumulation during cold acclimation has shown significant positive correlations with plant low-temperature tolerance (Kishor et al., 2005; Shang et al., 2011; Theocharis et al., 2012). Parallel to the rise in proline content, the metabolic pathway changes as well. *P5CS* and *ProDH* gene expression has been observed to be significantly increased under low temperature stress, and the level of *P5CS* displayed trends similar to those for proline content, indicating that proline accumulation occurred *via P5CS* and *ProDH* cooperation (Chen et al., 2014b; Koenigshofer and Loeppert, 2019). However, studies have also suggested that the OAT and PDH enzymes appear to be determinants of proline accumulation in plants under cold shock (Ruiz et al., 2002). In this research, the expression levels of *OsP5CS2*, *OsOAT*, and *OsProDH1* were all significantly increased, and the Glu and Orn concentrations were both significantly reduced (**Figures 2** and **3**), which suggests that proline accumulation resulted mainly from the cooperation of both the Glu and Orn pathways. This is consistent with a previous study on drought stress (Yang et al., 2015).

Mechanisms by which mycorrhizal plants counteract stress are mediated through biochemical changes that assist in the escalated secretion of osmolytes such as proline. The constructive correlation between AMF-mediated cold tolerance in host plants and proline accumulation has been addressed extensively by many researchers (Zhu et al., 2010; Chen et al., 2014a). The higher proline content in AM plants suggests that the plants inoculated with AMF have better capacity for osmotic adjustment than do non-mycorrhizal plants in the presence of abiotic stress. A similar result was obtained in this research: AMF colonization could significantly increase the accumulation of proline under low temperature, suggesting the greater resistance of mycorrhizal rice to low temperature compared to that of non-mycorrhizal plants (**Figure 1**). Hence, mycorrhizal rice exhibited more favorable growth parameters than did non-mycorrhizal rice under low temperature (**Table 1**). In contrast, in several studies, a decrease in proline accumulation was observed in mycorrhizal plants in comparison with non-mycorrhizal plants, leading researchers to conclude that plants colonized by AMF suffer less damage due to drought and evade stress adversities through lower proline contents (Ruiz-Sanchez et al., 2010; Chun et al., 2018). Proline accumulation in less tolerant plants has also been linked to a progressive agent of tissue injury as an accompanying consequence of NaCl exposure (Ruiz-Lozano et al., 2012). The rice in this research is a low-temperature-sensitive species and has a lower capability to resist stress. Consequently, AMF promote rice proline accumulation as a strategy for improving rice low-temperature tolerance.

Glu can be considered the “Center of the Universe” for N metabolism in plants, since most assimilated N passes through this compound before it is redistributed to major N metabolites (Forde and Lea, 2007). The metabolism of Glu into Orn, arginine, proline, and polyamines forms a major network of N-metabolism pathways in plants, and most of these amino acids play important roles under stress conditions (Majumdar et al., 2016). AMF colonization significantly increased the accumulation of Glu at normal temperature, while mycorrhizal rice contained a lower Glu concentration when suffering low-temperature stress. The results of this study also showed that AMF had little effect on Orn accumulation under the two temperature conditions (**Figure 2**), suggesting that AMF might increase Glu flux toward Orn/Arg and proline. Actually, the network of Glu metabolism is very complex, and depletion of Glu caused by increased synthesis of other amino acids may be partially compensated by its increased biosynthesis from assimilated N (Majumdar et al., 2016). The roles of AMF in its metabolism still need systematic studies, especially under stress conditions.

One report has suggested that stress-induced higher proline concentrations are accompanied by an increase in P5CR and P5CS activity and a decrease in OAT activity, indicating that the increase in proline accumulation in mycorrhizal seedlings may be associated with AMF-modulated upregulation of Glu synthetic pathways but not the Orn synthetic pathway (Wu et al., 2017). Additionally, studies have shown that AMF colonization lowers proline accumulation by downregulating the Glu pathway (Zou et al., 2013). Data from the present study reveal that AMF inoculation triggered higher proline concentrations under low temperature accompanied by elevated *OsP5CS* and *OsOAT* gene expression levels, suggesting that proline accumulation in mycorrhizal symbiont under low temperature was derived from the enhancement of both the Glu and Orn synthetic pathways (**Figure 3**). Thus, symbiotic proline metabolism under stress conditions remains controversial and may be related to the external environment and plant and fungal species.

Sufficient ATP production is crucial to plant growth and AM symbiosis establishment. Our previous research showed that the production of ATP in non-mycorrhizal rice decreased significantly under low-temperature conditions, whereas mycorrhizal rice contained the same ATP level as under normal temperature. Therefore, symbionts uphold energy levels and support plant growth under low-temperature stress (Liu et al., 2015). The degradation of proline yields high energy, approximately 30 ATP equivalents per molecule (Liang et al., 2013). Hence, mycorrhizal symbiont can also provide rich energy supplementation in terms of proline accumulation. Under low temperature stress, the *OsProDH1* gene was significantly upregulated by AMF colonization, demonstrating that higher ATP was generated *via* proline catabolism in mycorrhizal rice (**Figure 3D**). Combined with the results described above, it can be concluded that the mycorrhizal rice exhibited greater proline accumulation *via* enhancing both the Glu and Orn pathway and proline catabolism under low temperature stress.

NO is an important product of N metabolism and regulates key protective metabolite levels in plants *via* enzymatic and transcriptional modulation of proline biosynthetic pathways (Filippou et al., 2013). NO could promote proline accumulation through the upregulated Glu or Orn pathway or both pathways and thus improve plant stress tolerance (Wang et al., 2013). In the present study, treatment with a NO donor significantly increased rice proline concentration and had a greater promotion effect on mycorrhizal rice, which showed a further increase under low-temperature stress (**Figure 1**). Accompanied by the accumulation of proline, the *OsP5CS*, *OsOAT* and *OsProDH1* gene expression levels were all induced by NO application (**Figure 3**), demonstrating that NO enhanced both the Glu and Orn pathways of proline synthesis and the process of proline catabolism in mycorrhizal symbionts. During the early stage of mycorrhizal symbiosis, host plant roots can be stimulated to release NO as well (Calcagno et al., 2012). Our previous studies also observed that rice plants inoculated with AMF increased NO content, and NO could act as an important signaling molecule in the process of AMF-improved low-temperature tolerance (Wang et al., 2013). Consequently, NO not only participates in the process of symbiont establishment but is also involved in AMF regulation of host plant proline metabolism under low-temperature stress.

The promotion of plant growth by AMF is dependent on the nutritional status of the environment, and fungal colonization of the host plant’s roots is generally inhibited when environmental nutrients are abundant. However, the contribution of AMF was found to be greater, while the plant roots could not absorb the nutrients they normally require, when the nutrient supply was inadequate (Schmidt et al., 2011; Nouri et al., 2021). The same results were observed under low-temperature stress, in which mycorrhizal symbiosis with low N supplementation provided a greater increased colonization frequency, resulting in better conditions for N and carbon metabolism (Wang et al., 2013). In this study, the promotion of proline metabolism induced by AMF colonization under normal- and low-temperature stress was affected by N level as well. AM seemed to provide greater contribution to proline accumulation under high N at normal temperature. However, under low-temperature stress, there was no significant difference between non-mycorrhizal and mycorrhizal rice in proline concentration at high N level, whereas mycorrhizal rice contained higher proline under low N condition (**Figure 4A**). AMF colonization at low N level also significantly decreased the Glu content under low temperature, while no difference was observed at high N level (**Figures 4B, C**). These results demonstrate that AMF inoculation-enhanced proline accumulation might upregulate the Glu pathway at low N condition. However, the enzyme activity and gene expression of proline metabolism in mycorrhizal rice under different N levels was absent in this study and remained to be explored in the next step.

N application level affects N metabolism, and the activities of NR, GS, and GDH have been found to be considerably lower under low-nitrate supply than under high-nitrate supply (Cruz et al., 2004; Liao et al., 2019). Similarly, we found that an increase in N application significantly increased NR and GS activities under both normal- and low-temperature stress (**Figure 6**). Inoculation by AMF was also able to increase NR and GS activities and seemed to show greater enhancement at low N level under low-temperature stress. In addition to be a key enzyme for N metabolism, NR is an important substrate of NO synthesis as well. Under low-temperature stress, AMF enhanced the NOS activity and then increased the NO content more significantly at low N levels (**Figure 5**). Combined with the results of colonization rate and plant growth parameters (**Figure 7**), these data suggest that the increased accumulation of NO induced by AMF colonization under low N level enhanced the establishment of symbionts and meanwhile regulated proline metabolism by enhancing the Glu synthetic pathway, subsequently exerting stronger resistance to low-temperature stress.

In conclusion, AMF promoted proline accumulation and then enhanced rice resistance to low-temperature stress, in which AMF mainly upregulated both the Glu and Orn synthetic pathways and enhanced proline degradation to accelerate rice proline metabolism. NO not only enhanced the establishment of mycorrhizal symbiosis but also played important signaling roles in mycorrhizal proline accumulation under low temperature by regulating the metabolism of proline. However, the promotion of proline accumulation by AMF under low temperature was affected by the N application level, with AMF having a greater contribution during this process with NO involved under low N level.

## Data availability statement

The original contributions presented in the study are included in the article/supplementary material. Further inquiries can be directed to the corresponding author.

## Author contributions

XP and ZL designed the research. ZL, SB, and JM wrote the manuscript. SB and TL did the experiments. XP, PL, and CY revised the manuscript. All authors contributed to the article and approved the submitted version.

## Funding

This work was supported by the Natural Science Foundation of Heilongjiang (YQ2021C015), National Natural Science Foundation of China (NSFC) (No. 41701290).

## Conflict of interest

The authors declare that the research was conducted in the absence of any commercial or financial relationships that could be construed as a potential conflict of interest.

## Publisher’s note

All claims expressed in this article are solely those of the authors and do not necessarily represent those of their affiliated organizations, or those of the publisher, the editors and the reviewers. Any product that may be evaluated in this article, or claim that may be made by its manufacturer, is not guaranteed or endorsed by the publisher.
